# Transbronchoscopic cryotherapy for a giant sputum plug straddling the tracheal carina: A case report

**DOI:** 10.1097/MD.0000000000046753

**Published:** 2025-12-26

**Authors:** Shuman Rang, Qiaoju Li

**Affiliations:** a Department of Painless Endoscopy, Jining No. 1 People’s Hospital, Jining, China.

**Keywords:** bronchoscopic cryotherapy, case report, giant sputum plug

## Abstract

**Patient concerns:**

A 69-year-old male patient, who has had laryngeal cancer for 24 years and is currently in a tracheostomy state, was recently diagnosed with esophageal cancer one month ago. After one cycle of chemoradiation, the patient visited the clinic complaining of breathlessness for the past two days.

**Diagnoses:**

A chest computed tomography scan revealed a strip-shaped, columnar, and high-density lesion in the lumen of the trachea as well as in the right and left main bronchi, which was initially thought to be secreted. Histopathological examination confirmed the presence of a sputum plug characterized by mucus components, inflammatory exudates, and necrotic tissue.

## 1. Introduction

Sputum plugging is a common complication in patients who have undergone tracheotomy after laryngeal cancer surgery. Long-term tracheotomy results in the loss of the upper respiratory tract’s ability to warm, moisten, and filter air. This leads to dry airways and impaired ciliary movement, resulting in the production of thick sputum that can easily obstruct the airway. Patients often require repeated airway humidification and artificial suction to manage these plugs.^[1]^ Additionally, when tracheotomy is performed alongside esophageal cancer treatment, particularly with chemoradiation, patients may develop radiation bronchitis.^[2]^ This condition can increase mucus production, further increasing the risk of obstruction of the airway by sputum plugs.

Bronchoscopy is the preferred method for the direct diagnosis and treatment of foreign bodies in the airway. When used in conjunction with forceps, loops or baskets, it can effectively improve the success rate of foreign body removal.^[3]^ Bronchoscopic cryotherapy is widely utilized for various applications, including reducing airway tumors, removing foreign bodies, and clearing blood clots and mucus from the airway.^[4]^ However, there are currently no reports of cases in which the trachea to the carina was completely obstructed by sputum plugs and subsequently cleared using this technique.

This case report describes a rare case of a giant sputum embolus riding across the tracheal carina that was successfully removed quickly by an electronic bronchoscopic freezing technique and discusses its clinical features and therapeutic strategies through a literature review.

## 2. Case presentation

**Figure 1.**
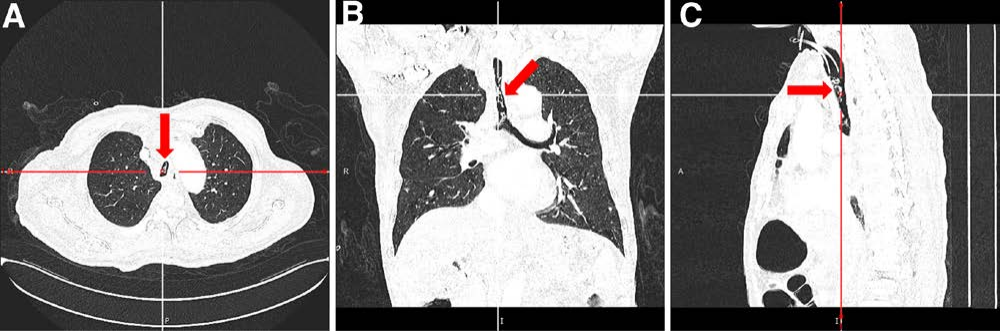
(A–C) Chest CT upon admission revealed multiple strip-shaped and columnar high-density lesions in the lumen of the trachea and the right and left main bronchi (red arrow). CT = computed tomography.

The patient is a 69-year-old male who presented with breathlessness lasting 2 days. He has a 24-year history of laryngeal cancer, for which he underwent surgery, and he is currently using a tracheostomy tube. One month ago, he was diagnosed with esophageal cancer and received radiotherapy, followed by 1 cycle of treatment with oxaliplatin, capecitabine, and tirilizumab at another hospital. Two days prior to this presentation, the patient experienced episodes of suffocation and wheezing without an obvious cause, accompanied by difficulty in coughing up sputum. Upon physical examination, his vital signs were as follows: temperature 36.4°C, heart rate 110 beats per minute, respiratory rate 24 breaths per minute, and blood pressure 176/116 mm Hg. Auscultation revealed clear respiratory sounds in both lungs, with no dry or wet rales or chest friction sounds. The peripheral blood oxygen saturation was 98%. A chest computed tomography scan revealed a mass in the lower esophagus. Additionally, the lung window of the chest computed tomography images displayed a strip-shaped and columnar high-density lesion located in the lumen of the trachea and the right and left main bronchi, leading to noticeable stenosis in the lumens of the trachea and main bronchi (Fig. 1), which is best shown in Figure 1B.

**Figure 2.**
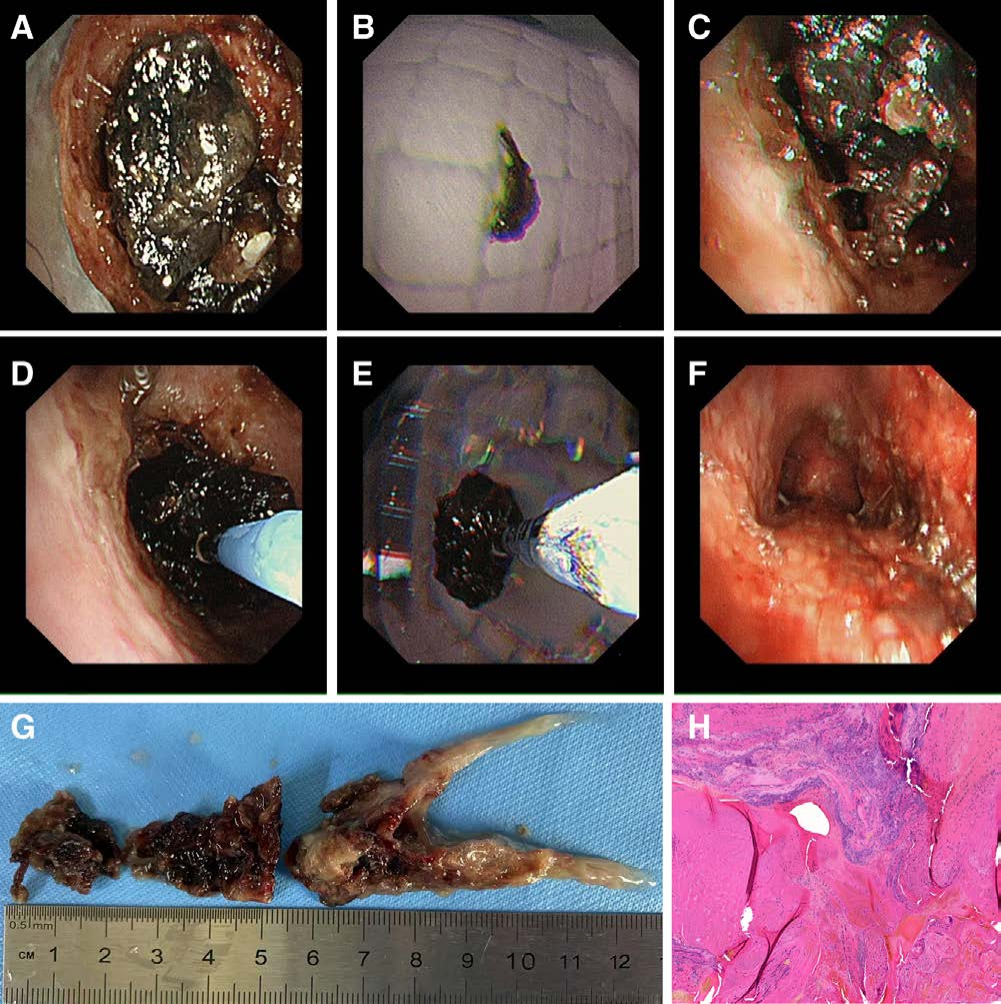
(A) Bronchoscopy revealed a black foreign body that completely blocked the tracheal lumen with the lumen narrowed by more than 90%. (B, C) A small amount of hard black mass was removed using crocodile forceps (jaw diameter 0.6 mm) (B), leaving most of the foreign body still lodged in place (C). (D) After freezing for 20 s, the condenser probe and the foreign body solidified into 1. (E) The condenser probe, foreign body and bronchoscope were removed together. (F) After the foreign body was removed, the main tracheal lumen was unobstructed, and the tracheal mucosa was diffusely congested and edematous. (G) Image of the entire foreign bodies. (H) The H&E staining images of the foreign bodies.

Electronic bronchoscopy was performed on the patient under general anesthesia. After entering the bronchoscope through the tracheal cannula, a black, lumpy foreign body was observed microscopically, completely blocking the tracheal lumen (Fig. 2A). The lumen was narrowed by more than 90%, making it impossible to advance the scope any further. Attempts to move the foreign body by pulling it back after clamping were unsuccessful, and applying a slight force caused it to break apart. Only a small amount of the hard black mass could be removed using the crocodile forceps (jaw diameter 0.6 mm) (Fig. 2B), leaving most of the foreign body still lodged in place (Fig. 2C). Given that repeated clamping would consume significant time and increase the surgical risk, as well as the potential for fragmented pieces to fall into the distal bronchioles, possibly leading to obstructive pneumonia and pulmonary atelectasis, we decided to utilize cryotherapy for the swift and complete removal of the foreign body. Since the foreign body was too large to pass through the tracheal tube, we removed the patient’s tracheal tube and directly accessed the site through the tracheotomy. Initially, 20 mL of saline was sprayed at the site of the foreign body blockage through the bronchoscope 3 times to wet and soften the foreign body. Next, a condenser probe was positioned near the foreign body and frozen for 20 seconds to solidify both the probe and the foreign body (Fig. 2D). The condenser probe was then removed along with the foreign body and the bronchoscope (Fig. 2E). This cryotherapy procedure was completed in 3 sessions, resulting in the extraction of a hard black mass of approximately 12 cm (Fig. 2G). Notably, the lower end of the foreign body removed in the last session was bifurcated, coinciding with the bifurcation of the main bronchus (Fig. 2G). Following the removal of the foreign body, repeat bronchoscopy revealed a clear lumen of the main trachea. At the same time, there was diffuse congestion and edema of the tracheal mucosa, with no active bleeding or neoplasm observed (Fig. 2F). Upon further examination, more pus was noted in the lumens of the right and left main bronchi, prompting endoscopic suction. The mucosa both in the main bronchi appeared mildly congested and edematous, with a significant amount of purulent secretion present; thus, microscopic suction was administered. Additionally, an alveolar lavage was performed in the lower lobes of both lungs and the upper lobe of the right lung for pathological examination. The patient’s symptoms of breathlessness were significantly reduced following operation. Their vital signs were as follows: heart rate was 82 beats per minute, respiratory rate was 19 breaths per minute, blood pressure was 103/72 mm Hg, and peripheral oxygen saturation was at 100% (with an oxygen flow rate of 2 L/min). The foreign body was sent for pathological analysis, which revealed a composition of mucus, inflammatory exudate, and necrotic tissue (Fig. 2H).

## 3. Follow-up

**Figure 3.**
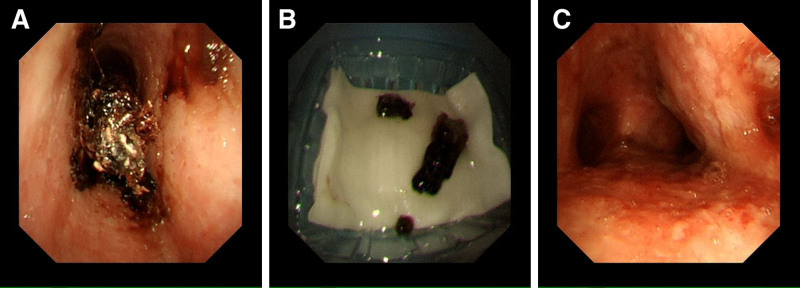
(A) Bronchoscopy revealed a dark brown foreign body that blocked the tracheal lumen. (B) Sputum plugs removed by cryotherapy. (C) After the sputum plugs were removed, the tracheal lumen was clear and unobstructed.

Four days after discharge, the patient underwent radiotherapy and chemotherapy for esophageal cancer at another hospital. On the evening of the day of radiotherapy and chemotherapy, the patient experienced shortness of breath again and returned to our hospital for a bronchoscopic cryo-embolectomy. Under the microscope, dark brown masses were observed in the tracheal lumen (Fig. 3A). On the following day, these black masses were removed during a bronchoscopy (Fig. 3B). The second episode of sputum plugging was approximately 5 cm. Unfortunately, the patient did not undergo a pathological examination for financial reasons. Repeat bronchoscopy revealed that the main tracheal lumen was not obstructed, although the mucosa appeared congested and edematous (Fig. 3C). The patient’s breathlessness disappeared after the procedure. Following discharge, the patient continued to receive radiotherapy and chemotherapy for esophageal cancer. During the treatment, nebulization and other symptomatic treatments were strengthened (the specific treatment plan was not known), and the patient did not experienced any wheezing symptoms during follow-up. Therefore, the rapid reformation of sputum plugs may be attributed to the increased secretions caused by radiation bronchitis, and the lack of adequate nebulization therapy to clear these secretions.

## 4. Discussion

Previous studies have shown that, in addition to causing radiation pneumonia and radiation pulmonary fibrosis, chest radiotherapy can also lead to radiation bronchitis.^[2]^ Radiation bronchitis results in mucosal congestion, edema, exudation, and ciliary dysfunction. This is particularly problematic for patients with a long-term tracheotomy, as the upper respiratory tract loses its ability to heat, moisten, and filter the air. Consequently, the airway becomes dry, and ciliary movement is impaired, making it easier for sputum plugs to obstruct the airway. Once sputum plugs are formed, they can obstruct airflow, leading to an imbalance between ventilation and blood flow. This imbalance may result in dyspnea, hypoxemia, and potentially acute respiratory failure. Furthermore, it can cause secretions to accumulate in the distal bronchi, thereby increasing the risk of lung infections. Prolonged retention of sputum plugs can also exacerbate airway mucosal congestion and edema, and even lead to erosion and bleeding, which increases the risk of suffocation. Timely identification and emergency treatment of airway obstructions caused by sputum plugs are essential. Bronchoscopy serves as the most intuitive diagnostic method to identify foreign bodies in the airway, including sputum emboli. Additionally, bronchoscopic intervention is the most direct and effective treatment option, with various available treatment modalities.^[5]^

Bronchoscopic cryoprobe is a safe and effective method for removing blood clots, mucus secretions, plastic bronchitis lesions, and foreign bodies. This technique involves freezing the foreign body together with the cryoprobe, allowing for removal regardless of the size, surface texture, or fragility of the object. It is particularly useful for extracting fragile and difficult-to-grasp items such as kernels, pills, teeth, chicken bones, mucus plugs, and necrotic tissue, which cannot be effectively clamped with traditional forceps.^[4]^ The operating principle involves the rapid release of high-pressure CO_2_ gas through a small orifice, which causes throttling expansion and cooling. This process forms an ice ball at the tip of the cryoprobe, reaching temperatures as low as ‐80°C.^[4,6]^ This method is particularly sensitive to water-containing tissues. For foreign bodies that contain little water, saline can be sprayed onto the object before freezing it with a cryoprobe. Sputum plugs are usually loose and fragile, making it difficult to grasp them entirely using conventional foreign body forceps in a single attempt. Multiple attempts may be necessary for complete extraction in cases with larger or harder sputum plugs. Notably, the frequent insertion and withdrawal of the bronchoscope can exacerbate mucosal damage, extend the duration of the procedure, and increase the risks associated with anesthesia.

In this case, the patient underwent a tracheotomy for laryngeal cancer. One month after receiving radiotherapy and chemotherapy for esophageal cancer, the patient developed a lung infection accompanied by significant sputum retention. A large sputum plug, measuring 10 cm was formed in the main airway and straddled the tracheal carina. This resulted in severe airway obstruction that was difficult to clear through the patient’s coughing, conventional tracheal suction, or drug expectoration. Since conventional methods for removing the sputum plug failed, we decided to use the bronchoscopic freezing method. During the procedure, physiological saline was infused to completely humidify the sputum plug. This increases the water content of the plug, enhancing the sensitivity of the bronchoscope to its presence. Additionally, the humidification softened the sputum plug and weakened its adhesion to the tracheal wall, which reduced the risk of mucosal bleeding and perforation while mechanically peeling it off the trachea. Research indicates that in nonemergency situations, specifically in patients 2 weeks post-hemostasis, the blood clots that form tend to be sticky and immobile. Therefore, it is advisable to apply short-term freezing extraction (for 3–4 seconds) to break the clots into smaller pieces, allowing them to be removed via suction and repeated freezing.^[7]^ Previous studies on the cryoextraction of acute, life-threatening airway clots have suggested using longer freezing times (between 15 and 120 seconds) to effectively remove the clot as a whole.^[1,7]^ In the case of our patient, who experienced significant suffocation, we opted for a freezing time of 20 seconds. During follow-up after discharge, the patient underwent additional radiotherapy and chemotherapy 4 days after the initial thrombectomy, leading to a recurrence of sputum plugging that evening. Based on our previous experience, we successfully removed the sputum plug using bronchoscopic cryotherapy. Following discharge, the patient continued with radiotherapy and chemotherapy while actively implementing measures such as nebulization to prevent further sputum plug formation. Since then, the patient has not experienced any suffocation.

In conclusion, this report presents what we believe to be the first successful removal of a large sputum plug using cryotherapy. Our case highlights the importance of maximizing the wetting and sensitivity of the condenser to the sputum plug when using cryotherapy to remove a rigid or giant sputum plug from the main airway. This method notably reduces the risk of bleeding and perforation during the stripping of the wet sputum plug. Additionally, we must remain vigilant for the formation of sputum emboli when administering chest radiotherapy to patients with a tracheostomy, as radiation can lead to radiation tracheitis. We will continue to monitor these patients and gather more experience in our clinical practice.

## Author contributions

**Investigation:** Shuman Rang.

**Methodology:** Shuman Rang.

**Project administration:** Shuman Rang.

**Supervision:** Qiaoju Li.

**Validation:** Qiaoju Li.

**Writing – original draft:** Shuman Rang.

**Writing – review & editing:** Qiaoju Li.
